# DiME: A Scalable Disease Module Identification Algorithm with Application to Glioma Progression

**DOI:** 10.1371/journal.pone.0086693

**Published:** 2014-02-11

**Authors:** Yunpeng Liu, Daniel A. Tennant, Zexuan Zhu, John K. Heath, Xin Yao, Shan He

**Affiliations:** 1 School of Computer Science, University of Birmingham, Birmingham, United Kingdom; 2 School of Cancer Sciences, University of Birmingham, Birmingham, United Kingdom; 3 Centre for Systems Biology, School of Biological Sciences, University of Birmingham, Birmingham, United Kingdom; 4 College of Computer Science and Software Engineering, Shenzhen University, Shenzhen, China; University of Torino, Italy

## Abstract

Disease module is a group of molecular components that interact intensively in the disease specific biological network. Since the connectivity and activity of disease modules may shed light on the molecular mechanisms of pathogenesis and disease progression, their identification becomes one of the most important challenges in network medicine, an emerging paradigm to study complex human disease. This paper proposes a novel algorithm, DiME (**Di**sease **M**odule **E**xtraction), to identify putative disease modules from biological networks. We have developed novel heuristics to optimise Community Extraction, a module criterion originally proposed for social network analysis, to extract topological core modules from biological networks as putative disease modules. In addition, we have incorporated a statistical significance measure, B-score, to evaluate the quality of extracted modules. As an application to complex diseases, we have employed DiME to investigate the molecular mechanisms that underpin the progression of glioma, the most common type of brain tumour. We have built low (grade II) - and high (GBM) - grade glioma co-expression networks from three independent datasets and then applied DiME to extract potential disease modules from both networks for comparison. Examination of the interconnectivity of the identified modules have revealed changes in topology and module activity (expression) between low- and high- grade tumours, which are characteristic of the major shifts in the constitution and physiology of tumour cells during glioma progression. Our results suggest that transcription factors *E2F4*, *AR* and *ETS1* are potential key regulators in tumour progression. Our DiME compiled software, R/C++ source code, sample data and a tutorial are available at http://www.cs.bham.ac.uk/~szh/DiME.

## Introduction

With the increasing availability of high-throughput, genome-wide assay data and high-performance computational resources, network biology (systematically reviewed by Barabási in [1]), which addresses the intrinsic structure and organisation of networks of pairwise biological interactions, has rapidly evolved as a promising research area. Viewing the functional machinery of the cell as a complex network of physical and logical interactions rather than a simple assembly of individual functional components has contributed unprecedented insight into the cell's wiring scheme.

The implications of methodology in network biology have been taken a step further by network medicine which focuses on the application to the understanding of complex disease pathophysiology [2]. The fundamental hypothesis is that the impact of genetic and environmental disturbance upon disease phenotype is likely to be asserted through coordinated activity of a group of genes and their products which interact intensively, termed as disease modules [2]. It has been argued that there is a significant overlap among the topological module (e.g., highly interlinked local region in the network), the functional module (e.g., a group of molecular components responsible for a particular cellular process), and the disease module consisting of disease-associated genes. A primary objective in network medicine, therefore, is to integrate the topological modules of biological networks and functional annotation to identify disease modules that contain both known and unknown disease genes and potential therapeutic targets.

To identify disease modules with high confidence, the first and most important step is the identification of significant and robust topological modules in a network constructed from patient data (e.g., gene co-expression network built from tumour microarray data). Several module identification algorithms was previously applied. One of the most popular algorithms is community detection algorithm that maximises a modularity measure brought forth by Newman (2006) [3]. Though it is capable of yielding biological insight in several case studies (e.g. [4] [5] [6]), a major drawback of the community detection algorithm is the resolution limit problem [7] [8] which results in huge modules with large numbers of genes (e.g., in [5]). Such problem is serious in disease module identification since it will inevitably introduce a lot of false disease genes (hence low specificity) and consequently adds difficulties to validation and interpretation.

Another popular algorithm is Molecular Complex Detection (MCODE) [9], which only identifies the nodes that actually belong to a module. It was originally developed to discover protein complexes in PPI networks but was extended to analyses of other network types (e.g., [10]). The key idea of the MCODE algorithm is to weight each node in the network with the minimum degree of the most densely connected set of nodes in its neighbourhood multiplied by the local density of that set, and recursively include neighbouring nodes into a module according to a user-tunable weight threshold starting from the highest weighting node. MCODE in general generates smaller and denser modules than the community detection algorithm does, but has the drawback that it only considers local connectivity, i.e., the links inside a module but ignores the links outside, which might generate biased results towards disease modules that contain genes or proteins with lots of interacting partners [11].

The community extraction (CE) algorithm is a novel community structure identification algorithm originally proposed for social network analysis [12]. This algorithm extracts the tightest module at a time, regardless of whether the rest of the network contains other modules. The algorithm is based on a novel module criterion, called community extraction (CE) criterion, which defines core modules in a network to be groups of nodes that are as densely connected as possible within the group while as loosely connected as possible to the rest of the network. This module criterion is very attractive for disease module identification because, unlike community detection, it will not result in huge modules. Moreover, in contrast to MCODE, it takes into consideration both the local connectivity of the module and its relationship to the global topology of the entire network. However, we found that in the original CE algorithm, the tabu search algorithm [13], [14], which is used for optimising the CE criterion, is not scalable to handle medium and large networks, hampering its application to disease module identification from biological networks which commonly consist of thousands of nodes.

In this paper, we propose a novel Disease Module Extraction (DiME) algorithm based on the CE criterion. Previously, we proposed a evolutionary community extraction algorithm and applied it to medium scale low and high grade glioma protein-protein interaction networks [15]. In order to handle large-scale biological networks, our DiME algorithm introduces a novel search heuristics using a simple local moving algorithm and a sample-and-seed step to prioritize candidate modules. Our algorithm has the advantage of good scalability (quadratic in time with respect to the network size), better accuracy and robustness than existing methods, and having few parameters to tune. In addition, we incorporated a statistical significance measure - the B-score as defined by Lancichinetti et al. [16], [17] - into the module extraction workflow to assess the quality of extracted modules without having to simulate large numbers of random networks for 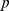-value calculation.

After identification of topologically and statistically significant modules, it would then be relatively straightforward to overlay biological annotations from multiple sources, such as Gene Ontology, transcription factor binding databases (e.g., the HTRI database) and literature reported disease genes (e.g., from the GeneCards catalogue) onto the modules to reveal key regulatory processes in disease and prioritize possible disease modules.

As a case study we have applied DiME to gliomas (glial tumours of the central nervous system). A large percentage (60%) of low grade (grade II) glioma patients have relatively long survival length of 5 years [18]. However, some patients may progress to more aggressive high grade (grade IV or GBM) glioma, termed Glioblastoma multiforme (GBM), which has a short survival length of approximately 15 months [19]. Although GBM has been intensively studied, the molecular mechanisms that underpin the progression from low to high grades gliomas still remain unclear. We have applied our DiME algorithm to two co-expression networks constructed from high- and low-grade glioma patient data to extract statistically significant modules. We then have compared the topology and activity (expression) of the disease modules, their functional annotations and regulatory mechanisms, to gain insights into molecular mechanisms in the acquisition of more aggressive malignancy during glioma progression. We have identified several statistically significant modules which are reproducible across three different datasets as potential disease modules. We then discovered that the dynamic activity, e.g., gene expression levels of these disease modules correlated with glioma progression. Finally from these disease modules we identify their upstream transcription factors *E2F4*, *AR* and *ETS1* as potential key regulators in tumour progression.

## Methods

### The DiME framework

**Figure 1:**
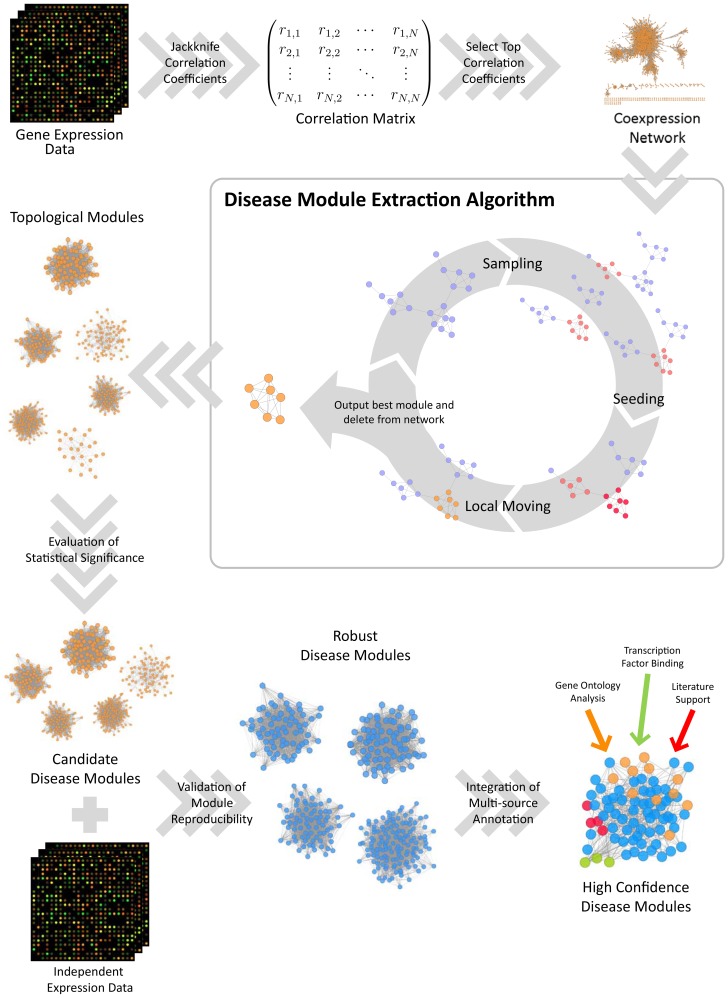
General work flow for the DiME framework.

A general work flow of the DiME framework for disease module identification and analysis is given in Figure 1. Note that our framework is readily adaptable to other types of study. For example, the construction of co-expression networks may be replaced by PPI networks to examine protein complexes or signaling modules, and the procedures downstream of the statistical significance evaluation step may also be varied according to specific aims of research, e.g. validation of disease modules via prediction of patient recovery/survival instead of correlating with tumour grade in our case study. In the following sub-sections, we provide details for the core steps of the DiME work flow - network construction, module extraction algorithm and evaluation of statistical significance.

### The DiME algorithm

Our DiME algorithm, as summarised as pseudo-code in Table 1, aims at maximizing the following objective function for community (termed as “module” throughout this paper) extraction defined in [12]:
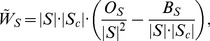
(1)where 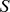 and 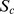 denote a module and its background network, respectively. 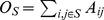, 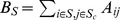 and 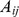 is the adjacency matrix. 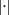 denotes cardinality. Intuitively the criterion seeks to maximise the density of connections within a module and minimise that with the rest of the network.

**Table 1:** Algorithm 1. DiME algorithm.

**Require**: 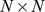 adjacency matrix 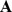 , number of initial solutions to be used ( 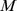 )
**repeat**
Create 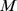 empty solutions (binary vectors) 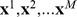 of length 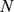
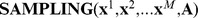
Create empty real-valued vector 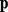 of length 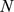
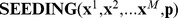
Create 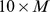 empty solutions 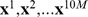 of length 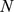
**for** 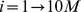 **do**
**for** 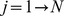 **do**
**if** 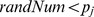 **then**
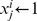
**else**
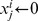
**end if**
**end for**
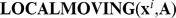
**end for**
Return solution with highest 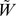
Delete current best solution (module) from network and update 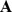
**until** highest 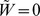

Maximizing the above objective function is essentially a combinatorial optimization problem, where each solution 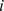 can be represented as a binary vector of 0 s and 1 s that denote the module membership status of each node:
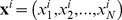
(2)where
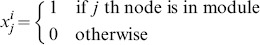
and 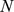 equals the total number of nodes in the network.

Finding the exact optimal solution for the problem takes exponential time. Therefore in the original publication [12], a generic metaheuristic algorithm, e.g., tabu search was used to solve the problem. Similarly, other metaheuristic algorithms such as evolutionary computation can be used [15]. However, from our experience, these generic metaheuristic algorithms suffer from scalability issues, e.g., when network size grows larger the time required for extracting a module increases disproportionately and quality of the extracted modules deteriorate significantly. This scalability issue greatly hinders the application of module extraction to biological network analysis where most of the networks consist of thousands of nodes.

In this paper, we propose a simple greedy local search algorithm that efficiently handles large networks. At each iteration, the algorithm visits all nodes in a sequential order. For each node, the algorithm performs the best move, e.g, flip the membership status of the node if it increases 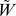. The algorithm iterates until no 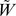-increasing move is found for any node. In order to speed up the algorithm, we only calculate the changes in the value of 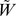:
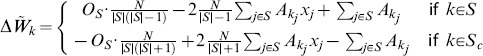
where 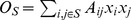. The detailed derivation of 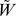 is provided in Section S3 in File S1. The local moving algorithm is summarised as pseudo-code in Table 2.

**Table 2:** Algorithm 2. Local moving function.

**function LOCALMOVING**(binary vector **x**, adjacency matrix **A**, problem size **N**)
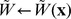
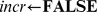
**repeat**
**for** 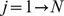 **do**
**if** 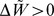 **then**
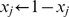
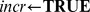
**end if**
**end for**
**until** 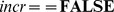
**end function**

However, our greedy local search algorithm will be trapped by local minima and the initial starting point is crucial to its performance. We propose a sample-and-seed approach to guide the greedy local search that both speeds up the search process and obtains better optima than the commonly used methods (data not shown). As shown in Table 3 and Table 4, the approach consists of two distinct stages of optimization: a sampling stage and a seeding stage. In the sampling stage, a small number of solutions are optimized using our local greedy search algorithm mentioned above, resulting in a set of locally optimal solutions. Note that at this stage no prior information about the size distribution of modules in the network is available, thus a gradient-like probability for each node being “1” is used, i.e., probabilities ranging from 0 to 0.5 are used evenly among the solutions. The probability is capped at 0.5 as we assume that for large biological networks it is unlikely that a meaningful module would cover more than half of the entire network. The optimized solutions are then passed to the second stage of the algorithm to estimate probabilities for each node being the “seed” of a module, which are then used to initialize a new set of solutions for optimization.

**Table 3:** Algorithm 3. DiME sampling function.

**function SAMPLING**(binary vectors 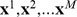 of length 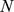 , network adjacency matrix 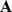 )
**for** 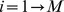 **do**
**for** 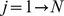 **do**
**if** 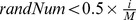 **then**
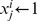
**else**
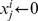
**end if**
**end for**
**end for**
**for** 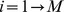 **do**
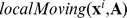
**end for**
**end function**

**Table 4:** Algorithm 4. DiME seeding function.

**function SEEDING**(real vector 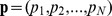 , solutions from sampling 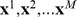 )
**for** 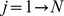 **do**
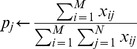
**end for**
**end function**

The estimation and seeding process used in our algorithm is relatively simple and straightforward: since our DiME method only extracts a single best module at a time, and by definition such a module should be a connected subgraph of the entire graph, we could for each extraction procedure view each node as a possible “seed” for the module to be extracted, which will progressively include its surrounding nodes to form the module during optimisation. The initial extraction with relatively few individuals would, then, act as the seed prioritizer. The probability of each node becoming the seed is naturally designed to be proportional to the frequency it appears in the initial solutions (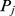 denotes probability of node 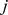 becoming the seed):
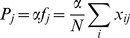
(3)Additionally, when viewed as a probability mass function (PMF) where each node position corresponds to a certain (possibly zero) probability of being the seed, the probabilities of the nodes being the seed should also sum to one:
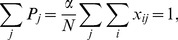
(4)which yields
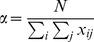
(5)Plug 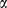 into equation 3, the above probabilities 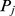 could be estimated by
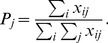
(6)These probabilities are then used to randomly seed a set of solutions. For the 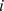
th node, a random number 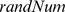 is generated and compared with 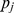, if 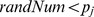, then 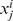 is seeded as 1, otherwise 0. Repeat this for all 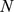 nodes to obtain solution 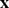. We construct 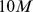 solutions and optimise them using local moving. After all 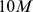 rounds of local moving, the best solution that emerges will be returned.

To extract all possible modules from the network, a sequential extraction procedure is used where each extracted module is deleted from the network before extracting the next one, until no more modules can be extracted from the network (i.e., best 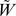 becomes 0). In all following analyses only modules with size larger than 2 were considered valid.

### Evaluation of the statistical significance of extracted modules

To ensure that the modules extracted from the biological networks are statistically significant, i.e. they are significantly different from modules that arise from random networks of an appropriate null model, we incorporated a B-score significance measure as proposed in [16] [17] as a quality control step for the modules. The B-score measure assumes a null model where edges within the module (community) of interest is held unchanged while the remaining connections in the network are randomly shuffled. Then the B-score is calculated based on the null module to quantify how often we should expect to see the module “by chance”. The B-scoring measure has a major advantage of avoiding large amounts of resampling cycles for simulating null model results. In our later experiments, we also showed that the B-scoring measure worked well with our DiME algorithm to detect statistically significant modules.

For details of the B-score calculation, the reader is referred to the original works [16] [17]. In order to make this paper self-contained, we provide the full procedure for B-score computation in Section S1 in File S1. In this study all B-score calculations were based upon default parameters in the original work with 20 independent runs for each module evaluation.

### Data acquisition and preprocessing

Raw expression data of 97 WHO grade II glioma patient and 126 glioblastoma (GBM) samples was downloaded from the NCI Rembrandt database [20]. The expression data was collected using Affymetrix Human Genome U133 Plus 2.0 microarrays (54,675 probe sets in total). Raw expression (.CEL files) was preprocessed and normalized using standard Robust Multi-array Average (RMA) [21] procedures in R and filtered for probe sets with duplicate Entrez ID mappings, no Entrez IDs or low variance in expression values (in this case lower 50% quantile of inter-quartile ranges). The resulting expression matrix contained 9,971 genes.

Two independent sets of brain tumour data for validation: the TCGA GBM dataset and grade II glioma expression dataset from the Gene Expression Omnibus database (GSE30339) [22], each consisting of 197 and 23 samples respectively (low-grade glioma data sources are relatively scarce) - were downloaded from the respectively online data repositories. The validation sets used the Affymetrix HG-U133A arrays (22,277 probe sets in total), different from the Rembrandt dataset. The downloaded datasets were already preprocessed and normalized with standard RMA [21]methods, and were subsequently filtered using R for non-specific binding with the same method as described above for the Rembrandt datasets. Preprocessing of the microarray data resulted in a total of 6,247 genes.

### Glioma co-expression network construction

For samples in each tumour grade, pair-wise Pearson's correlation coefficient (PCC) was calculated for each gene pair to generate the correlation matrix of all genes. In order to guard against possible outliers, a jackknife [23] [24] approach was used to estimate the true gene expression correlation coefficients. The raw PCC values, 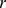, were first converted to 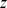 values using Fisher transformation [23]:
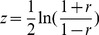
(7)


The (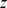-transformed) jackknife correlation value for any given gene pair 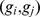 (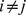), 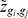, is calculated as follows:
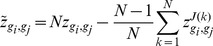
(8)where 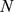 is sample size and 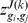 is 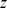-transformed PCC between genes 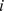 and 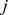 calculated with the 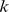
th sample excluded.

To construct a co-expression network, similar to the construction method used in [25], we ranked all gene pairs according to their absolute values of the jackknife correlation values 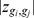. We then selected the a certain percentage of the top ranking gene pairs as significant co-expressions, which will be connected as a co-expression network. This percentage, called network construction threshold in our paper, will affect the edge noise level of the resulting network. For example, a stringent threshold will miss large numbers of true edges while a larger threshold will introduce many false-positive edges. For our glioma co-expression network analysis, we set the network construction threshold to 0.1%. In the Results section, we also present data regarding how different network construction threshold values, therefore different noise levels, affect module extraction results of the DiME algorithm.

Our main analyses will be focused on the Rembrandt grade II glioma and GBM datasets described in Data Acquisition and Preprocessing, and datasets from other sources will be used for result validation. The resulting networks for the Rembrandt datasets contained 2,739 (GBM) and 3,888 (grade II glioma) nodes (genes) respectively. The networks have 49,705 edges for both GBM and grade II glioma networks. The resulting networks both followed good power-law degree distribution, with power-law fit correlation of 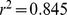 (GBM) and 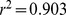 (grade II glioma) respectively.

## Results

### The DiME algorithm has better accuracy and scalability than the original CE algorithm

Since biological benchmark networks are scarce, we chose four social networks, which have been widely used as benchmarks in many previous studies, to evaluate the accuracy and scalability of our DiME algorithm in comparison with the original CE algorithm. In addition, these four benchmark networks also covered a wide range of size and complexity and are thus ideal for evaluating the scalability of DiME. These benchmark networks include: 1) a university e-mail network [26], referred to as the Email network; 2) the Erdös collaboration network among mathematicans [27], referred to as the Erdös network; 3) a network of users of the pretty good privacy (PGP) algorithm for secure information transactions [28], referred to as the PGP network; 4) the relationships between authors that shared a paper in condensed matter physics [29], referred to as the Cond-mat network. The basic characteristics of the network are listed in Table 5.

**Table 5:** Characteristics of the benchmark networks.

	Network Name			
Algorithm	Email	Erdös	PGP	Cond-mat
No. of Nodes	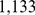	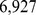	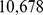	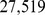
No. of Edges	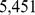	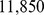	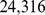	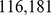

We ran the DiME algorithm to extract the tightest module (i.e., module with highest 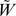 in the network) in each network and repeated the extraction for 50 times to calculate the mean and standard deviation of 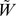 and computation time. We compared our DiME algorithm with the original CE method which is based on the tabu search algorithm. In our experiments, we used a tabu list size of 10, and for each independent run the algorithm stopped when the highest 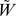 ever achieved did not increase in 300 consecutive iterations. The choice of tabu list size, ranging from 2 to 100, did not affect the general output (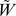, data not shown), and a choice of 300 iterations in the stopping criterion is a compromise between computational overhead and full convergence of the algorithm. All these experiments were performed using single CPU threads.

The statistics for 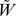 and computation time were shown in Tables 6 and 7, respectively. Note that no data was shown for the Cond-mat network using original CE algorithm as it took several hours even for a single run which made multiple runs infeasible.

**Table 6:** 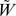
 scores of the first module of each benchmark network.

	Network Name			
Algorithm	Email	Erdös	PGP	Cond-mat
DiME	**14420.04**±22.76	**103544.8**±2.32	**401530.9**±6274.66	**3032925**±0
Original CE	**12967.58**±14.18	**103587.5**±79.22	**385675**±3681.49	-

The results in bold font indicate the they are statistically significant (Student's 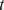-tests 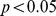).

**Table 7:** Computation time (second) for extracting the first module in each benchmark network.

	Network Name			
Algorithm	Email	Erdös	PGP	Cond-mat
DiME	**0.915**±0.104	**30.837**±2.419	**54.436**±2.705	**350.920**±23.567
Original CE	**1.219**±0.246	**162.023**±641.856	**463.916**±364.553	-

The results in bold font indicate the they are statistically significant (Student's 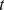-tests 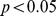).

It can be seen from Table 6 that in general our DiME algorithm outperforms the original CE algorithm in terms of accuracy as it is capable of locating better maxima of 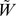 for the Email and PGP networks with relatively low variation in the 50 trial runs. DiME is also more scalable than the original CE algorithm as it consumes significantly less average computation time with much smaller standard deviations, as shown in Table 7.

### Parameter setting of the B-score cutoff

One parameter that needs to be tuned in DiME is the statistical significance (B-score) cutoff for extracted modules. As the B-score cutoff becomes smaller, e.g., more stringent, more modules and thus more genes including disease genes would be discarded, decreasing the sensitivity of DiME. Vice versa, when the B-score cutoff becomes larger, large number of non-disease genes will be included which reduces DiME's specificity.

In order to balance the specificity and sensitivity of DiME, we carried out experiments to find the optimal value of B-score cutoff. Since the B-score is based on null distribution probabilities and may thus be viewed as the widely used statistical 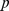-values, here we evaluated the loss of genes under three most commonly used levels of statistical significance cutoff - 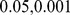 and 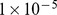. The results for all datasets used in this paper (see Data Acquisition and Preprocessing in Methods for dataset specifics) are shown in Table 8.

**Table 8:** Relative loss of genes under different B-score cutoffs.

	B-score Cutoff		
Algorithm	0.05	0.001	1 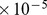
Rembrandt Data (**GBM**)	**32.97%** (574/1741)	**50.09%** (872/1741)	**54.68%** (952/1741)
TCGA Data (**GBM**)	**30.19%** (358/1186)	**42.50%** (504/1186)	**51.85%** (615/1186)
Rembrandt Data (**grade II Glioma**)	**47.27%** (1230/2602)	**62.95%** (1638/2602)	**68.14%** (1773/2602)
GEO Data (**grade II Glioma**)	**42.46%** (1106/2605)	**66.64%** (1736/2605)	**71.48%** (1862/2605)

Table 8 shows that in general 50%–70% of the genes identified by the DiME algorithm belong to modules with B-score statistical significance level of 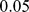, 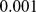 and 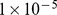. The percentage of retained genes experienced a large decrease at a B-score cutoff of 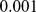, but dropped more smoothly at a further increase in the stringency of cutoff. Observe that the grade II glioma datasets show a larger loss of genes than the GBM datasets at the same cutoff, probably due to the relatively scarcer low-grade glioma samples and possibly higher tumour heterogeneity in the sample cohort. It seems that 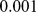 is a reasonable value for the B-score threshold where relative loss of genes stops increasing dramatically. We used this 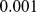 as our default value throughout our experiments.

### Statistical significance measure B-score correlates with module extraction criterion 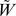


**Figure 2:**
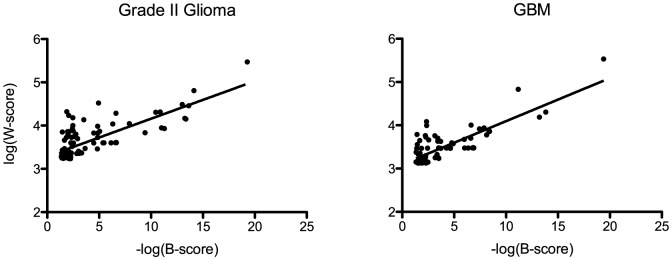
Correlation of 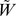 scores with B-scores. All modules with size larger than 2 and B-score 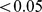 are included. A few modules whose B-score is 0 (indicating scores exceeding the lower limit of detection in the B-score algorithm) were excluded. Fitted lines of 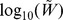 versus 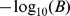 are shown. The fitted Pearson's correlation 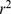 values are 0.57 (grade II glioma, left panel) and 0.65 (GBM, right panel) respectively, with both correlation 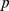 values smaller than 0.0001 in Pearson's correlation tests.

In order to investigate the relationship between Statistical significance measure B-score and module extraction criterion 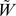, we applied our DiME algorithm to extract all modules from the Rembrandt grade II glioma and GBM networks. We excluded modules identified by DiME with size smaller than 2 genes. We calculated the Pearson's correlation between B-score and 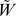. As shown in Figure 2, the B-score of statistically significant (B-score 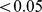) modules extracted from both the glioma networks is well correlated with the value for the CE criterion, 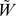 (Pearson's correlation test 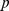-values smaller than 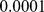), indicating that the CE criterion is likely to be built upon a null model which fits well with that assumed by the B-score measure.

### DiME identifies more significant modules than the community detection algorithm

Using Rembrandt glioma networks, we carried out experiments to compare the performance of DiME with the community detection algorithm [30]. It is worth mentioning that although the community detection algorithm essentially partitions the network into modules, which is very different from our DiME algorithm and therefore difficult to compare with, it is still interesting to investigate which algorithm is better at identifying biological relevant disease modules.

We executed the community detection algorithm on the Rembrandt networks to partitioned the two networks into 131 and 105 modules, respectively. However, we found that the largest module identified by the community detection algorithm consisting of 1,372 genes out of a total of 3,888, and is statistically non-significant under the B-score scheme (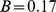). A careful inspection of this large module shows that three of the statistically significant (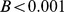) modules extracted by DiME, with sizes of 212, 39 and 42 genes respectively, are contained or almost contained within it (i.e., over 90% overlap with the large module). It also has significant overlaps with several other non-significant DiME modules. Such an observation suggests that community detection is not appropriate for disease module identification in large biological networks, since it generates huge modules with large numbers of genes of different functions, which adds difficulties to validation and interpretation. Based on the results, we exclude community detection for comparison with DiME in the subsequent experiments.

### DiME is more robust for identifying significant modules from noisy co-expression networks than MCODE

As discussed in the network construction section, the network construction threshold for selecting significant co-expressions as edges significantly affects the edge noise level of the resulting network. In this sense is the DiME algorithm able to robustly capture the most essential (“*core*”) topological components of the network against different levels of edge noise? In other words, will the modules extracted by DiME differ significantly when the network noise level is altered?

In order to evaluate the robustness of DiME, we define a conservation score, which essentially quantifies the similarity between the modules extracted from a noisy network and those extracted from a reference network, which can be viewed as a ground-true network without any edge noise. We chose the two Rembrandt networks of grade II glioma and GBM with a network construction threshold of 0.1% as the reference networks for comparison since they are the networks that were used in our further analyses. The details of the calculation of the conservation score is in Section S2 in File S1. We then constructed noisy networks with different levels of edge noise by changing the network construction thresholds of the reference networks to 0.5%, 0.2% and 0.05%. The DiME algorithm was then applied to each of these networks to identify all modules for the calculation of the conservation score. Box plots of the distribution of scores across modules in a network were plotted. We also compared the popular MCODE algorithm [9] with DiME using the same experiments.

**Figure 3:**
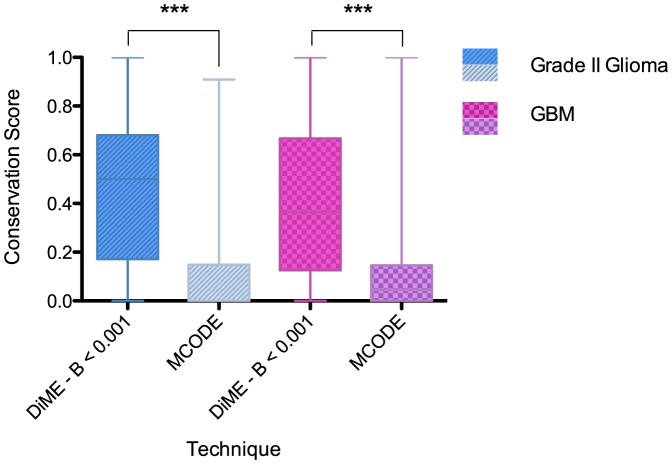
DiME is robust to edge noise in co-epxression networks. Shown in the plots are results for the grade II glioma networks (left panel) and GBM networks (right panel). The horizontal axes display the technique used, and vertical axes show average conservation scores. Only modules with size larger than 5 are taken into consideration. Asterisks denote statistical significance in Student's 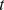-tests when comparing means with MCODE modules: “***” - 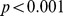.

As shown in Figure 3, the conservation scores of DiME modules were significantly better (Student's 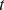-tests 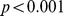) than those of MCODE modules across networks constructed with the same set of genes but different edge noise levels. Such robustness is further strengthened by the fact that under all B-score cutoffs the DiME algorithm extracts more nodes in total than does MCODE, and that loss of nodes in DiME modules was not very dramatic even under very stringent B-score cutoffs (See Table S1 in File S1).

### Module extracted by DiME from Rembrandt Grades II and GBM networks are biologically relevant to glioma progression

**Figure 4:**
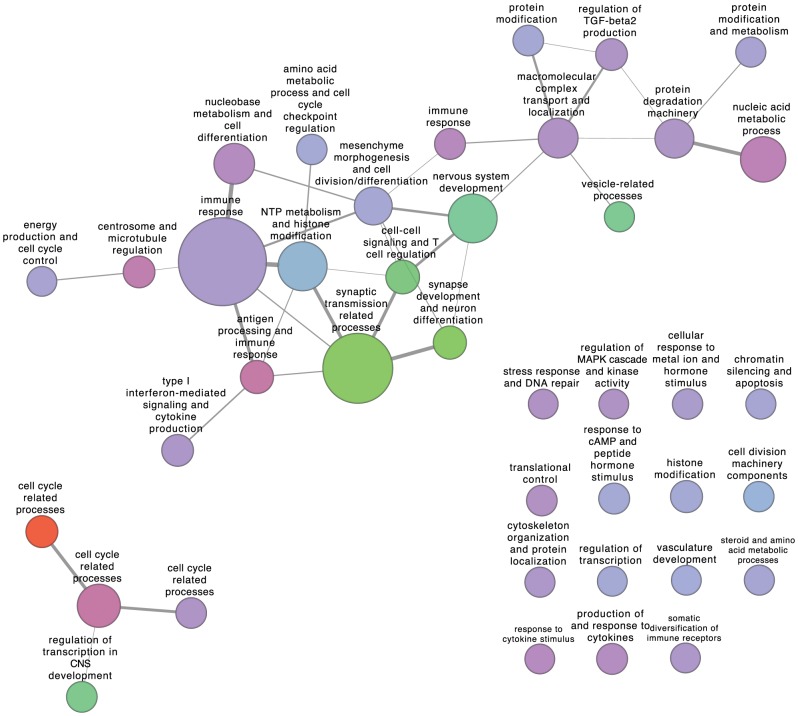
Visualisation of grade II glioma modules with B-score less than 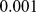 and their inter-module connectivity. Nodes represent extracted modules, node size represents module size and node color represents (log-transformed) fold-change in average module gene expression level compared with normal patient samples (Red - increase in average expression, green - decrease in average expression, lavender - no change in average expression). Edge widths are proportional to connectivity (i.e., number of co-expression gene pairs) between module pairs.

**Figure 5:**
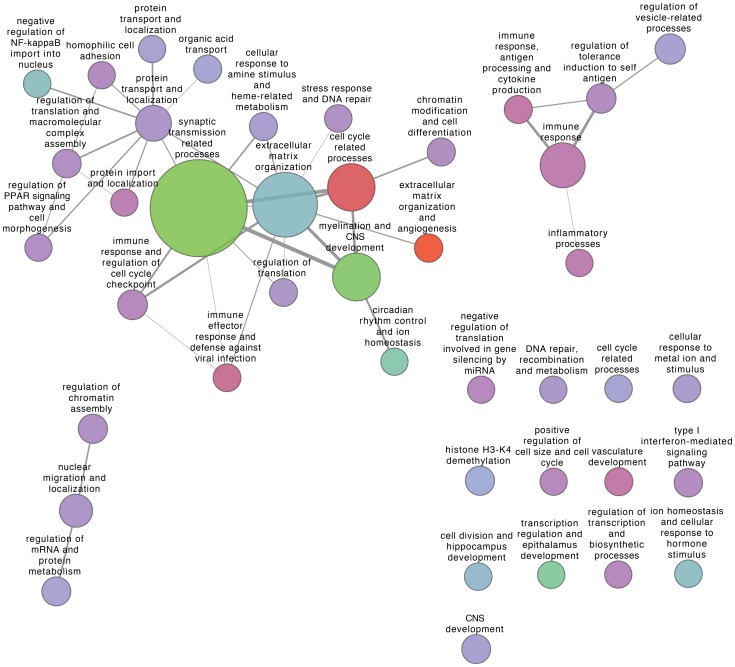
Visualisation of GBM modules with B-score less than 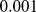 and their inter-module connectivity. Nodes represent extracted modules, node size represents module size and node color represents (log-transformed) fold-change in average module gene expression level compared with normal patient samples (Red - increase in average expression, green - decrease in average expression, lavender - no change in average expression). Edge widths are proportional to connectivity (i.e., number of co-expression gene pairs) between module pairs.

We applied the DiME algorithm with a B-score cutoff of 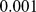 to the two Rembrandt glioma datasets, and visualised the resulting modules and their interconnectivity in Figures 4 and 5. Each module is annotated with a specific function summarised from its enriched Gene Ontology terms (false discovery rate 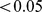 in hypergeometric tests). Edge widths are designed to be proportional to the number of connections (co-expression pairs) between two modules, in order to illustrate strength of coordination between functional components in the disease network. Node color represents fold change of average expression level of all genes in one module compared with normal patient samples.

The grade II glioma module network (Figure 4) demonstrates a significant shift in the tumour phenotype compared with normal samples. As would be expected, there appears to be a marked down-regulation of normal neuronal function (i.e. synapse transmission-related processes), and a significant increase in cell cycle-associated processes. It is of interest to note that the modules associated with immune response are slightly, but significantly increased in grade II tumours.

As shown in Figure 5, progression to grade IV (GBM) is marked by a significant shift in network topology despite the general conservation of module functional annotation: inter-module connectivity was significantly altered in the GBM tumour network compared with that of grade II gliomas, with strengthened co-expression between cell cycle-related processes and ECM reorganisation and modules associated with differentiation status, such as synaptic transmission and CNS development. In addition, there was a breakdown in the co-expression of immune processes and the above mentioned modules. However, GBM tumours appear to have altered levels of transcripts involved in extracellular matrix (ECM) reorganisation and angiogenesis - markers of a more aggressive phenotype.

### Modules extracted by DiME from Rembrandt grade II and GBM networks are reproducible in independent datasets

To verify the reproducibility of the disease modules from the Rembrandt networks, we applied the DiME work flow to two independent sets of brain tumour data: a GBM dataset from the TCGA database, and a WHO grade II glioma expression datasets from the GEO database published by Turcan et al. [22], which used a different microarray chip from that used by the Rembrandt dataset (see Data Acquisition and Preprocessing in Methods for details). The aim of this experiment is to see if DiME can extract disease modules that reproducible in independent datasets. The same DiME work flow, i.e., disease co-expression network construction, module extraction and evaluation of statistical significance were performed using exactly the same methods and parameters as those for the Rembrandt dataset. We also employed MCODE for comparison. The same experiments and the same work flow except evaluation of statistical significance, i.e., B-score thresholding (see discussion) were applied.

Network construction resulted in a network with 3,635 nodes and 19,509 edges for the GEO grade II glioma expression data, and one with 1,787 nodes and 19,509 edges for the TCGA GBM data. The GEO grade II glioma data co-expression network had 1,617 nodes in common with the Rembrandt network (Jaccard index 0.2737), while the TCGA GBM network had only 717 nodes in common with the Rembrandt counterpart (Jaccard index 0.1882). We show that even in this situation where the two sets of glioma disease networks significantly differ from each other in gene ensemble, our DiME algorithm is still capable of reproducing modules with fairly similar composition.

Because classical methods for comparing graph clusterings, e.g., the adjusted Rand index or normalized mutual information [31], are designed for comparing partitioning of the same network, they cannot be used to evaluate the similarity between extracted modules of the Rembrandt dataset and those of the validation datasets. Here we score the reproducibility of each module from the Rembrandt networks (grade II glioma and GBM) using the following steps:

1. For each tumour grade, project all modules from both the Rembrandt and the validation (TCGA or GEO) network onto the intersection of all genes in the two networks, resulting 2 sets of projected modules. (Projection is calculated as intersection.)
2. For each projected module with size larger than 5 from the Rembrandt network, calculate its maximum possible Jaccard index with the projected modules (corresponding to a best-matching pair of modules) from the corresponding validation network and return the Jaccard index as its reproducibility score.

Note that we chose a module size threshold of 5 here to guard against random effects brought about by small modules. Such a threshold did not qualitatively affect comparison with the performance of MCODE over a reasonable range of 2–10 (data not shown).

**Figure 6:**
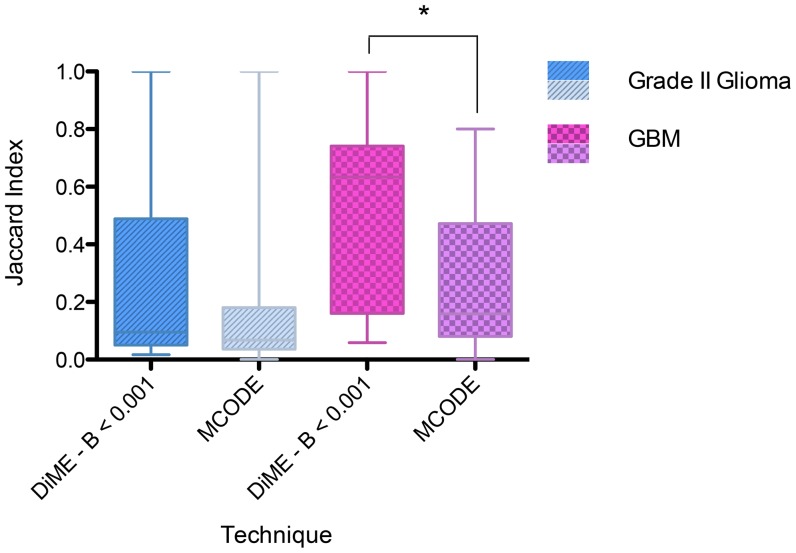
Comparison of module reproducibility among different algorithms. Shown are box plots of average reproducibility (Jaccard index) for each technique used. Asterisks denote statistical significance in Student's 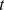-tests when comparing means with MCODE modules: “*” - 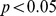.

The results are shown as box plots of Jaccard index distributions in Figure 6. Average Jaccard indices of 0.28 and 0.51 were observed for the grade II and GBM datasets respectively, showing a high level of module reproducibility for both tumour grades considering the remarkable differences in the microarrays. Inspection of Gene Ontology enrichment of modules in the independent datasets also showed that they are functionally similar to the matched modules in the Rembrandt counterpart (data not shown). It may be seen from the GBM data box plots that under stringent B-score cutoffs (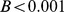) the upper quantiles of the Jaccard index distribution show markedly increased average values and decreased range of variation, compared with those of MCODE modules. The average Jaccard index for all DiME modules with 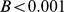 is also significantly higher than that of the MCODE modules (Student's 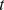-test, 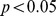) in the GBM datasets, and a similar trend, though not highly significant (Student's 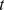-test, 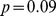), was observable for the grade II glioma datasets. Statistical insignificance may be attributed to the fact that the MCODE modules showed large variance in the Jaccard indices. It is noteworthy that the low-grade glioma data generally displayed considerably less reproducibility than that of the high-grade counterpart. This might be due to the relatively smaller sample size and possible heterogeneity in the samples (which might indicate existence of molecular subtypes across the patient cohorts).

### Expression levels of common modules shared by Grade II and IV gliomas are correlated with tumour grade

It is natural to expect that disease modules extracted from grade II and GBM sample datasets might be used to distinguish samples of different tumour grades. Such distinguishibility did not seem to be readily achievable solely at the functional level: GO Biological Process enrichment analysis of the two sets of modules obtained from the two tumour grades showed that they are enriched with highly similar functions, with the majority of modules in both grades functionally annotated with 1) immune response, 2) synaptic transmission, 3) cell cycle regulation, 4) nervous system development and/or 5) cell migration/adhesion, though the GBM modules seemed to have a larger portion annotated with immune response and cell cycle-related functions. We hypothesize that a combination of functional annotation and expression landscape of the common modules, however, may shed light upon the shifts in the major regulatory mechanisms responsible for tumour progression.

To test our hypothesis, we first matched functionally similar modules extracted from the Rembrandt grade II and GBM networks using a GO semantic similarity measure as used in [5]. Using this method, we obtained a pair-wise similarity matrix by calculating the GO semantic similarity measure between all pairs of modules with one module from the Rembrandt grade II glioma network and the other from the Rembrandt GBM network. Since the number of modules extracted from the GBM network and of those from the grade II glioma network are similar, best-matching module pairs may be easily found by the Hungarian algorithm for assignment problems [32] [33]. The above process resulted in 41 best-matching pairs (one-to-one mapping) of modules which were then intersected to yield 12 common modules shared by both tumour grades in the Rembrandt networks. We discard the modules with less than 5 genes to guard against possible artifacts of noise in data acquisition and/or network construction. The 12 modules were then projected onto the gene universe of the independent GEO grade II glioma and TCGA GBM networks to identify common modules that are conserved across two microarray types. We also excluded modules with less than 5 genes. The final set of common modules is comprised of 9 modules and 208 genes.

Two-tailed Jonckheere-Terpstra test was then performed to examine whether tumour grade was correlated with the expression signature of the 9 conserved modules. The expression signature of each common module was calculated as the average expression of all genes in the module. The test discovered 7 out of the 9 common modules (183 genes in total) whose expression signatures were significantly correlated with tumour grade (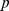 value 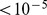 after adjusting for FDR control).

We carried out GO Biological Process enrichment analysis on the 7 common modules. As tabulated in Table 9, the 7 common modules are all significantly enriched with at least one GO BP term after Benjamini-Hochberg adjustment for false discovery rate (FDR 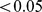). It is also interesting to see from the table that the functional annotations of these modules covered most of the summarised functions in the connected components of module inter-connectivity networks shown in Figure 4.

**Table 9:** Summary of functional annotation and location information of the conserved common modules.

Module Number	Top 3 GO BP Terms	Chromosome Locations	Transcription Factors
1	immune response ( 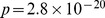 )immune system process ( 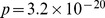 )regulation of immune system process ( 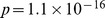 )	1, 2, 3, 4, 5, 6, 7, 8, 9, 10, 11, 12, 14, 16, 17, 19, 20, 21, 22, X	*AR*, *E2F4*, *EGR1*, *ETS1*, *GATA2*, *GATA3*, *YBX1*
2	synaptic transmission ( 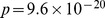 )multicellular organismal signaling ( 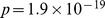 )cell-cell signaling ( 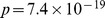 )	1, 2, 3, 4, 5, 6, 7, 8, 9, 10, 11, 12, 13, 15, 16, 17, 19, 20, 22, X	*AR*, *E2F4*, *ESR1*, *ETS1*, *FOXP3*, *GATA1*, *GATA2*, *HIF1A*, *MYC*, *YBX1*
3	nervous system development ( 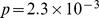 )myelination ( 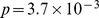 )ensheathment of neurons ( 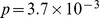 )	1, 2, 3, 4, 6, 7, 8, 9, 11, 12, 15, 16, 17, 19, X	*AR*, *ESR1*, *ETS1*, *GATA2*, *PRDM14*, *TFAP2C*, *YBX1*
4	ribonucleoside triphosphate catabolic process ( 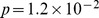 )purine ribonucleoside triphosphate catabolic process ( 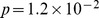 )positive regulation of growth ( 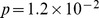 )	3, 6, 7, 8, 12, 14, 17, X	*AR*, *ESR1*, *ETS1*, *HIF1A*
5	antigen processing and presentation of exogenous peptide antigen via MHC class I, TAP-dependent ( 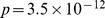 )antigen processing and presentation of exogenous peptide antigen via MHC class I ( 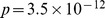 )antigen processing and presentation of peptide antigen via MHC class I ( 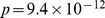 )	6	*E2F4*, *ETS1*
6	M phase ( 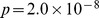 )cell cycle progress ( 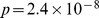 )nuclear division ( 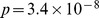 )	1, 4, 8, 10, 15, 17, 20	*AR*, *E2F4*, *ESR1*, *ETS1*
7	type I interferon-mediated signaling pathway ( 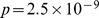 ) cellular response to type I interferon ( 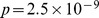 )response to type I interferon ( 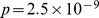 )	1, 2, 12, 21	*AR*, *E2F4*, *ETS1*, *GATA1*

**Figure 7:**
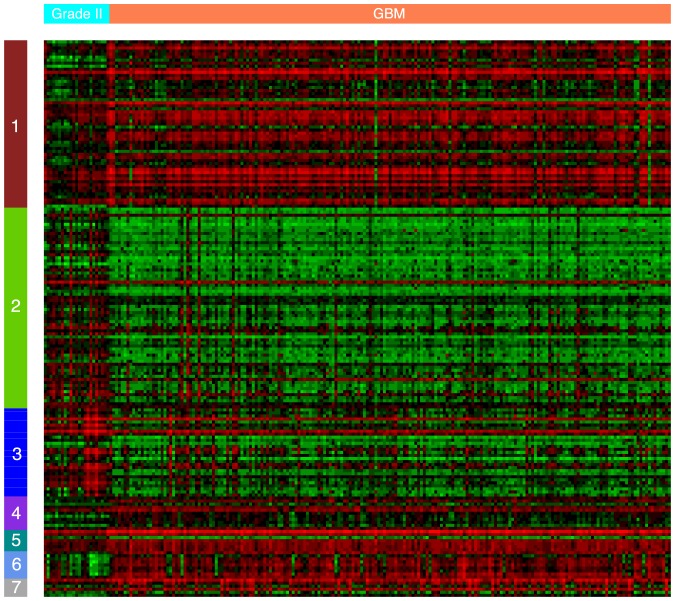
Heat map showing expression landscape of all genes in the 7 conserved common modules across grade II glioma and GBM samples. Rows correspond to genes grouped by modules and columns correspond to samples grouped by tumour grade.

Figure 7 shows a heat map of the expression level of individual genes in the 7 modules grouped by modules (rows) and samples by tumour grade (columns). The clear differential expression patterns of genes belonging to the same module across grades are easily observable in Figure 7. For example, activity of modules 1 and 7, corresponding to the regulation of immune response, increased with malignant progression - i.e. grade II to GBM. Taking into account that the expression arrays were performed on samples of the total tumour mass (not isolated glial cells), and the nature of the transcripts represented by the immune-associated modules, this may be a significant observation. We hypothesize that the significant loss of co-expression observed between the modules associated with cell cycle and glial differentiation and those involved in immune function is indicative of the infiltration of immune cells into the tumour mass in GBM samples. Indeed, this is in agreement with literature reports that have shown an increase in T cell infiltration into GBMs which is around 5 times more than that observed in grade II gliomas [34].

### Regulatory mechanisms underlying the common modules shared by grades II and IV

We also extracted the transcription factors that bind to the genes of each common modules from the Human Transcriptional Regulation Interactions database developed by Bovolenta et al. (2012) [35]. We summarise the results in Table 9. An intriguing observation is that the 7 common modules showed high similarity in their transcriptional regulators, as seen from the transcription factors that bind to genes in each module. All 7 modules are regulated by *ETS1*, which is involved in the control of stem cell development and often in tumorigenesis [36]–[38]. *E2F4*, a transcription factor that binds to and inhibits several tumour suppressor proteins, as well as induces DNA synthesis required for cell proliferation, is also shared by 5 modules. Another important cancer-associated transcription factor that is shared among the modules is *AR*, a steroid hormone receptor that regulates downstream processes such as proliferation and differentiation and whose mutation has been shown to play important parts in cancer [39]–[41]. The transcription factors *E2F4*, *ESR1*, *ETS1* and *MYC* are all downstream targets of the well-established tumour supressor gene *TP53* that is responsible for multiple alterations in the gene regulatory network in gliomablastoma [42]–[44]. These results suggest that the common modules identified through our method are likely to be downstream mediators of the effects of alterations to master regulators in glioblastoma-associated pathways.

### DiME Identifies Unique Biologically Relevant Modules Not Discovered by Other Methods

In order to investigate whether DiME can discover modules that cannot be identified by other algorithms, we compared all B-score significant (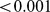) DiME modules to those identified by MCODE and the original CE algorithm. We defined a module to be missing if it has no corresponding modules showing an overlap of larger than 20% of the smaller module in comparison.

Our results showed that the original Tabu-search based CE algorithm, under the same 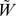 module criterion, failed to identify several both statistically significant and biologically meaningful coexpression modules in grade II glioma and GBM. Besides, the results were highly unstable across independent runs. Even when we looked at the best results (containing 7 and 6 modules with B-score 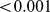 for grade II glioma and GBM respectively) we have so far obtained, the original CE algorithm still missed several of the statistically significant DiME modules shown in Figures 4 and 5, such as the large “immune response” module in grade II glioma and the “myelination and CNS development” module in GBM.

There is one module from each of the two grades that was identified by DiME method but missed by the MCODE method. As they were also missed by the original CE method, we view these modules as uniquely identified by DiME, and employ previously reported evidence to demonstrate their pathophysiological relevance. Both modules contain more than 10 genes and are thus non-trivial.

In the unique module identified by DiME from the grade II glioma network (corresponding to “mesenchyme morphogenesis and cell division/differentiation” module in Figure 4), we highlight the genes *ABCA5*, *RGN* and *MYC*, all among the top degrees of connectivity in the module. They encode a member of the ATP-binding cassette (ABC) sub-family 1 transporters, regucalcin and the Myc proto-oncogene protein, respectively. ABC transporters have been suggested to mediate drug sensitivity of subpopulations of cancer stem-like cells across many tumour types [45] [46] [47]. Regucalcin is a calcium-binding protein involved in calcium homeostasis and carbohydrate metabolism, and is recently reported as a newly identified tumour suppressor [48]. It is also not surprising for the module to include the well-established proto-oncogene *MYC* which has a wide spectrum of downstream effectors [49] [50], and the SLC family member *SLC13A3*, the expression of which found to be down-regulated in tumour cells over-expressing *MYC* family genes [51] [52]. Interestingly, *MYC* did not appear in the entire set of genes in MCODE modules. Decreased expression of one of *RGN*'s coexpression partner *SELENBP1* which encodes a selenium-binding protein, has also been shown to be associated with multiple tumour types [53] [54] [55].

In the unique module identified by DiME from the GBM glioma network, the *RRAS* oncogene, the SH3 domain binding kinase gene *SBK1* and the transcription factor *SOX8* involved in CNS development have the highest degrees of connectivity. *RRAS* regulates cell migration and has been identified as a glioblastoma multiforme signature gene [56] [57]. *SBK1* is dysregulated in multiple cancer types and may display a broad range of cellular functions [58]. *SOX8* has been shown to be predominantly expressed in oligodendrocytomas, astrocytomas and glioblastomas and may be an early glial marker for medulloblastomas [59]. In Figure 5 this module corresponds to the “regulation of vesicle-related processes” node as it contains several components involved in intra-cellular vesicle transport.

In conclusion, DiME algorithm identified two disease modules missed by the other two algorithms whose components were established targets of tumour treatment and/or key regulatory molecules in glioma. Gene members of the above two mentioned modules are provided in Tables S2 and S3 in File S1.

## Discussion

One major advantage of our DiME algorithm is that it is relatively fast, with worst case time complexity of 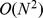. In general for a network of ∼7,000 nodes, it takes less than one second to fully optimise a single solution on a Core i7 computer using a single thread. Another advantage of the algorithm is its small number of parameters and robustness to varying parameters. The only user-specified parameter is the solution set size, and in most cases 50∼100 solutions should give satisfactory results for large networks.

Since optimization of individual solutions is independent of one another, the optimization process is readily parallelizable. In our implementation the publicly available OpenMP® [60] library for parallel computing on Intel® processors is used, and multi-core processor users can specify the number of parallelly processing cores to be used.

We have not only demonstrated that the new DiME algorithm outperforms the original Tabu search-based community extraction method in terms of speed and maxima of 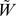 values, but also shown that the original method does not seem to be feasible for analysing coexpression networks even if it could handle the time complexity - the modules extracted by the original method were too large for interpretation, and contained unconnected nodes which are indicative of premature convergence.

An additional advantage of incorporating the B-score scheme into our DiME algorithm is that a simple hard-thresholding approach alone is sufficient to retain most of the large modules. Whereas modules with low statistical significance may be trimmed into significant ones using the OSLOM algorithm proposed by Lancichinetti et al. (2011) [17], such a procedure might be inefficient as the calculation of B-scores is quadratic in time with respect to module size and may become computationally expensive, especially for huge modules that arise from modularity-based community detection algorithms.

Note that while more than 45% of extracted genes were retained under the most stringent B-score cutoff used (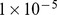), such robustness against statistical significance cutoffs was not observed for other algorithms such as MCODE and modularity-based community detection. Even at a less stringent B-score cutoff of 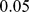, the MCODE and modularity-based modules would generally suffer from a loss of over 50% and 95% of identified genes, respectively (see Table S1 in File S1 for comparison). Therefore, we did not include the B-score significance measure for the MCODE modules in all comparative analyses.

The problem of resolution limit in community detection methods is also manifested in the size and statistical significance of modules. Using the Rembrandt grade II glioma data as an example, the largest module identified by the community detection method as of [30], consisting of 1,372 genes out of a total of 3,888, was deemed statistically non-significant under the B-score scheme (mean 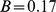). A careful inspection of this large module showed that three of the statistically significant (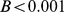) DiME modules (corresponding to immune response, macromolecular complex transport and localization and nucleobase metabolism and cell differentiation, see Figures 4 and 5), with sizes of 212, 39 and 42 genes respectively, are contained or almost contained within it (i.e., larger than 90% overlap with the large module). It also has significant overlaps with several other non-significant DiME modules. In comparison, three MCODE modules are contained within the above mentioned large module, with sizes of 77, 18 and 13 genes respectively (corresponding to immune responses, nucleic acid metabolism and regulation of cytoskeleton). Such an observation suggests that community detection is not appropriate for disease module identification in large biological networks, since it generates huge modules with large numbers of genes which add difficulties to validation and interpretation.

An analysis of the variability of module identification results show that core modular structure of the Rembrandt coexpression networks used in the case study is well conserved under varying network construction parameters (see Appendix, Figure 3). Such conservation is consistent with the concept of “module core” described by the original authors of module extraction [12]. It is worth pointing out, however, that the less conserved modules do not necessarily bear little functional significance in the network, as their fluctuations may be due to the noise in the biological data itself, rather than in the module identification algorithm. The construction of a highly robust network per se is still a highly active area of research and is not the main focus of this paper.

The module connectivity networks for grade II glioma and GBM samples provide a high-level yet insightful understanding of brain tumour progression and the associated rewiring of cellular machinery. A common expression signature of both tumour grades is down-regulation of nervous system development and normal neuronal functions (e.g., synaptic transmission) and up-regulation of cell cycle (cell proliferation) related progresses (Figures 4 and 5), light green and red nodes). Such concomitant alterations in transcriptome are consistent with a malignant phenotype - cells that are becoming less differentiated and are proliferating more. The coordination between the two types of functional processes is remarkably strengthened in GBM compared with grade II glioma samples (manifested in the increased coexpression links between the corresponding modules), a possible consequence of the significant increase in the transcription factors *AR* and *ETS1* shared by the two processes in both grades. Core components of the two processes are also conserved across microarrays, as is shown by the expression levels of modules 2, 3, and 6 in Figure 7.

Also of pathological significance is the significant increase in the activity of the angiogenesis-related module in GBM. The module is linked via coexpression to another module which is related to extracellular matrix organisation and controls cell morphology and physical interaction with its environment, in accordance with putative functions of extracellar matrix components (e.g., TGF 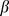-induced, encoded by *TGFBI* from the extracellular matrix organisation module) in promoting angiogenesis [61]. The increase in these modules as well as those representing cell cycle processes and the further decrease in modules associated with differentiation are indicative of a tumour that is becoming markedly more malignant with progression from grade II to GBM. As this analysis has shown that all of these processes are co-ordinately regulated, the identification of two transcription factors that are associated with all or almost all of these modules suggests that both *E2F4* and *ETS1* play a significant role in the pathogenesis of glioma.

Our results suggest that DiME could uncover statistically significant modules whose highly connected members have been found to be important biomarkers or key cancer regulators, as exemplified in the last section in Results. These modules were not found in the overlap of genes between DiME and MCODE modules, indicating the inherently different modular structures detected by the two methods. Though MCODE was able to identify genes such as *TGFB2*, a putative glioma tumour regulator and drug target [62] [63], they were mostly included in modules that displayed very low statistical significance (B-score close to 1), indicating a high likelihood of statistical artifacts. Because these individual candidate genes with weaker topological context but significant dysregulation in cancer are readily identifiable using single-gene analysis methods such as differential expression and copy number variation, we conclude that our DiME algorithm can be applied to biological networks in parallel with single-gene analysis for enhanced understanding of the overall shift in the cellular regulatory program in disease.

Taken together, the above discussed modules may be viewed as potential disease modules whose dynamic activity dictates tumour progression. The results show that the core methodology introduced in this paper, including the DiME algorithm and the accompanying B-score scheme for evaluating statistical significance, is capable of extracting modules of coordinately expressed genes that point to key regulators in disease networks and thus provide a more systematic understanding of complex disease progression.

## Supporting Information

File S1Supporting Information that contains description of the B-score Algorithm Pseudo-code, the calculation of the conservation score, the derivation of 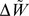 and two unique DiME modules found in Grade II and IV glioma coexpression networks.(PDF)
