# Supplementary material for: DiME: A Scalable Disease Module Identification Algorithm with Application to Glioma Progression
Source: PLoS One. 2014 Feb 11;9(2):e86693. doi: 10.1371/journal.pone.0086693 (PMC3921127; doi:10.1371/journal.pone.0086693)
Supplement: File S1 — Supporting Information that contains description of the B-score Algorithm Pseudo-code, the calculation of the conservation score, the derivation of and two unique DiME modules found in Grade II and IV glioma coexpression networks. (PDF) [file pone.0086693.s001.pdf]

Supporting Information for DiME: A scalable disease module identification algorithm with application to glioma progression

Yunpeng Liu[1], Daniel A. Tennant[2], Zexuan Zhu[4], John K. Heath[3], Xin Yao [1], Shan He[1,3*]
1 School of Computer Science, University of Birmingham, Birmingham, UK
2 School of Cancer Sciences, University of Birmingham, Birmingham, UK
3 Centre for Systems Biology, School of Biological Sciences, University of Birmingham, Birmingham, UK
4 College of Computer Science and Software Engineering, Shenzhen University, China
∗ **E-mail: s.he@cs.bham.ac.uk**

The B-score Algorithm Pseudo-code

Algorithm 1 The B-score Algorithm

function BSCORE(module C)
    $t \leftarrow 0, C_0 \leftarrow C, B_0 \leftarrow \varnothing, n_C \leftarrow |C|$
    while $t < n_C - 1$ **do**
        for each $i \in C_t$ **do**
            Calculate p_i as $p_i = \sum_{q=k_i^{int}}^{k_i} f(q|C)$
        end for
        $w_{t+1} \leftarrow$ vertex in C_t with highest p_i
        $w_{t+2} \leftarrow$ vertex in C_t with second highest p_i
        $B_{t+1} \leftarrow B_t \bigcup \{w_{t+1}\}$, $C_{t+1} \leftarrow C_t \setminus \{w_{t+1}\}$
        Recalculate p values for all nodes currently in B_{t+1}
        $p_l \leftarrow$ lowest p value of vertices in B_{t+1}
        if $p_{w_{t+2}} > p_l$ **then**
            swap($p_{w_{t+2}}, p_l$)
        end if
        Compute $Pr(< S_{t+1}|C_{t+1}, B_{t+1}, p_{w_{t+2}})$, where $S_{t+1} = \sum_{i \in B_{t+1}} p_i$
        $t \leftarrow t + 1$
    end while
    Return $\min_t Pr(< S_t|C_t, B_t, p_{w_{t+1}})$
end function

The B-score measure assumes a null model where edges within the module (community) of interest is held unchanged while the remaining connections in the network are randomly shuffled. A probabilistic measure based on hypergeometric distribution is then calculated for each module member to evaluate the likelihood that the observed number of within-module edges would arise from the null model. Such a probability is then summed over the number of possible within-module connections (from the observed value to the maximum possible value - the total degree of the node) to give a cumulative probability p_i of observing an intra-module degree equal to or larger than the observed value under the null model.

The above p_i is calculated for all nodes in the module and sorted to identify the "worst" node in the module - the node with the highest p_i. The B-score algorithm assumes that, for a truly non-random module, the probability of observing such a worst p_i value as the *minimum* among all nodes currently not belonging to the module is expected to be very low under the null model for its calculation. The original B-score algorithm also incorporated a stochastic element into the calculation of p_i, took into consideration a list of k worst nodes in the module and utilized the probability that the sum of the scores of these worst nodes in a module obtained from a random background model is smaller than the observed value as the final statistical significance measure (the B-score) for the module. The B-score is calculated over multiple runs and the average is used for evaluation of module statistical significance. Such an additional step has been shown to act as a resampling step and guard against possible significant community structure from random graphs.

Calculation of the conservation score

We first define a reference network which can be seen as the ground true network without noise. We extract the modules from the network as reference modules: R_i $(i = 1, \dots, n_r)$. We also define a set of m noisy co-expression networks by introducing m different levels of edge noise to the reference network.

We extract the modules N_{i_j} $(i = 1, \ldots, n_n)$ from the jth noisy network and repeat this for all m noisy networks. Then we perform the following steps to calculate the conservation score.

1. Find the best matching module N_{k_j} in the jth noisy network for the kth reference modules R_k, where k_j is obtained by:
$$k_j = \arg\max_i \frac{|R_k \bigcap N_{i_j}|}{\min(|R_k|, |N_{i_j}|)}$$
2. Repeat step 1 to find the best matching modules in all m noisy networks: N_{k_j}, $j = 1, \ldots, m$.
3. Calculate the conservation score for the reference module R_k using the following formula:
$$ConservationScore(R_k) = \frac{|R_k \bigcap_{j=1}^m N_{k_j}|}{|R_k|}$$
4. Repeat steps 1-3 to calculate conservation scores of all reference modules $ConservationScore(R_i)$, $i = 1, \ldots, n_r$.

Derivation of $\Delta\tilde{W}$

Let x_i be the boolean variable indicating whether the ith node is selected as a community member. Denote the entire set of nodes as V, and N as the total number of nodes in V. Let $\mathbf{A}$ be the adjacency matrix of the entire network. Following the denotations used by Zhao et al. (2011), we have

$$\tilde{W}_S = O_S \cdot \frac{|S_c|}{|S|} + O_S - (O_S + B_S) = \sum_{i,j \in S} A_{ij} x_i x_j \left(\frac{|S_c|}{|S|} + 1 \right) - \sum_{i \in S, j \in V} A_{ij} x_i \tag{1}$$

If node k is in S, the only move that will change $\tilde{W}$ is to move it from S to S_c. The new $\tilde{W}$ after moving will be:

$$\tilde{W}_{S'} = \left(\sum_{i,j \in S} A_{ij} x_i x_j - 2 \sum_{j \in S} A_{kj} x_j \right) \left(\frac{|S_c| + 1}{|S| - 1} + 1 \right) - \left(\sum_{i \in S, j \in V} A_{ij} x_i - \sum_{j \in V} A_{kj} \right); \tag{2}$$

and if node k is in S_c, the only move that will change $\tilde{W}$ is to move it from S_c to S. The new $\tilde{W}$ after moving will be:

$$\tilde{W}_{S'} = \left(\sum_{i,j \in S} A_{ij} x_i x_j + 2 \sum_{j \in S} A_{kj} x_j \right) \left(\frac{|S_c| - 1}{|S| + 1} + 1 \right) - \left(\sum_{i \in S, j \in V} A_{ij} x_i + \sum_{j \in V} A_{kj} \right) \tag{3}$$

Therefore, we can calculate the change in the value of $\tilde{W}$ when node k is in S:

$$\begin{aligned}
\Delta\tilde{W}_k &= \tilde{W}_{S'} - \tilde{W} \\
&= \left(O_S - 2 \sum_{j \in S} A_{k_j} x_j \right) \left(\frac{|S_c| + 1}{|S| - 1} \right) - O_S \cdot \frac{|S_c|}{|S|} - \sum_{j \in S} A_{k_j} x_j + \sum_{j \in S} A_{k_j} \qquad (4) \\
&= O_S \cdot \frac{N}{|S|\,(|S| - 1)} - 2 \frac{N}{|S| - 1} \sum_{j \in S} A_{k_j} x_j + \sum_{j \in S} A_{k_j}, \qquad (5)
\end{aligned}$$

Similarly, we can obtain $\Delta\tilde{W}$ when node k is in S_c

$$\begin{aligned}
\Delta\tilde{W}_k &= \tilde{W}_{S'} - \tilde{W} \\
&= \left(O_S + 2\sum_{j\in S} A_{k_j} x_j\right)\left(\frac{|S_c|-1}{|S|+1}\right) - O_S \cdot \frac{|S_c|}{|S|} + \sum_{j\in S} A_{k_j} x_j - \sum_{j\in S} A_{k_j} \qquad (6)\\
&= -O_S \cdot \frac{N}{|S|\left(|S|+1\right)} + 2\frac{N}{|S|+1}\sum_{j\in S} A_{k_j} x_j - \sum_{j\in S} A_{k_j}, \qquad (7)
\end{aligned}$$

Combine the above two equations, we finally derive the equation for $\Delta\tilde{W}$:

$$\Delta\tilde{W}_k = \begin{cases} O_S \cdot \frac{N}{|S|(|S|-1)} - 2\frac{N}{|S|-1}\sum_{j\in S} A_{k_j} x_j + \sum_{j\in S} A_{k_j} & \text{if } k \in S \\ \\ -O_S \cdot \frac{N}{|S|(|S|+1)} + 2\frac{N}{|S|+1}\sum_{j\in S} A_{k_j} x_j - \sum_{j\in S} A_{k_j} & \text{if } k \in S_c \end{cases}$$

where $O_S = \sum_{i,j\in S} A_{ij} x_i x_j$.
It is easy to observe from (9) and (10) that the change in $\tilde{W}$ for any flipping of node membership can be calculated in linear time (O_S itself can be updated in linear time for each flip too).

Two Unique DiME Modules in the Grade II and IV Glioma Co-expression Networks

Figure Legends

Tables

Table S1. Relative loss of genes under different B-score cutoffs

	Technique								
	DiME			MCODE			Modularity		
B-score Cutoff	0.05	0.001	$1{\times}10^{-5}$	0.05	0.001	$1{\times}10^{-5}$	0.05	0.001	$1{\times}10^{-5}$
Rembrandt Data (**GBM**)	**32.97%** (574 / 1741)	**50.09%** (872 / 1741)	**54.68%** (952 / 1741)	**58.09%** (452 / 778)	**81.36%** (633 / 778)	**83.03%** (646 / 778)	**41.16%** (1073 / 2607)	**49.33%** (1286 / 2607)	**99.04%** (2582 / 2607)
TCGA Data (**GBM**)	**30.19%** (358 / 1186)	**42.50%** (504 / 1186)	**51.85%** (615 / 1186)	**33.75%** (188 / 557)	**39.14%** (218 / 557)	**45.60%** (254 / 557)	**2.14%** (36 / 1681)	**45.63%** (767 / 1681)	**90.78%** (1526 / 1681)
Rembrandt Data (**grade II Glioma**)	**47.27%** (1230 / 2602)	**62.95%** (1638 / 2602)	**68.14%** (1773 / 2602)	**61.96%** (728 / 1175)	**65.79%** (773 / 1175)	**69.96%** (822 / 1175)	**94.97%** (3546 / 3734)	**98.23%** (3668 / 3734)	**98.93%** (3694 / 3734)
GEO Data (**grade II Glioma**)	**42.46%** (1106 / 2605)	**66.64%** (1736 / 2605)	**71.48%** (1862 / 2605)	**56.59%** (466 / 822)	**67.76%** (557 / 822)	**74.70%** (614 / 822)	**94.76%** (3255 / 3435)	**99.33%** (3412 / 3435)	**99.71%** (3425 / 3435)

Table S2. Module Members in A Unique DiME Module (Grade II Glioma) Larger than 10 Genes

Module Name	Gene Symbol	Gene Product
Mesenchyme morphogenesis and cell division / differentiation (grade II glioma)	*MEX3A*	Mex-3 RNA Binding Family Member A
	EBF4	Early B-Cell Factor 4
	HEY1	Hairy/Enhancer-Of-Split Related With YRPW Motif 1
	KCNQ2	Potassium Voltage-Gated Channel, KQT-Like Subfamily, Member 2
	SLC13A3	Solute Carrier Family 13 Member 3
	SLC22A15	Solute Carrier Family 22, Member 15
	ABCA5	ATP-Binding Cassette, Sub-Family A (ABC1), Member 5
	CAMKMT	Calmodulin-Lysine N-Methyltransferase
	PNPLA4	Patatin-Like Phospholipase Domain Containing 4
	RGN	Regucalcin
	ECHDC2	Enoyl CoA Hydratase Domain Containing 2
	PRMT5	Protein Arginine Methyltransferase 5
	MAML2	Mastermind-Like 2
	ZNF22	Zinc Finger Protein 22
	C16orf89	(Chromosome 16 Open Reading Frame 89)
	KLHL26	Kelch-Like Family Member 26
	CLQL1	Complement Component 1, Q
	KLRC3	Killer Cell Lectin-Like Receptor Subfamily C, Member 3
	S100A13	S100 Calcium Binding Protein A13
	SELENBP1	Selenium Binding Protein 1
	TPPP3	Tubulin Polymerization-Promoting Protein Family Member 3
	MYC	Proto-Oncogene C-Myc
	FAM50B	Family With Sequence Similarity 50, Member B
	CHST6	Carbohydrate (N-Acetylglucosamine 6-O) Sulfotransferase 6
	RYR3	Ryanodine Receptor 3
	RCAN3	Calcipressin-3
	PHLDA1	Pleckstrin Homology-Like Domain, Family A, Member 1
	SULF2	Sulfatase 2
	RPLP0	Ribosomal Protein, Large, P0
	CDKL2	Cyclin-Dependent Kinase-Like 2 (CDC2-Related Kinase)
	C11orf96	Chromosome 11 Open Reading Frame 96
	STPG1	Sperm-Tail PG-Rich Repeat Containing 1

Table S3. Module Members in A Unique DiME Module (Grade IV Glioma) Larger than 10 Genes

Module Name	Gene Symbol	Gene Product
Regulation of vesicle-related processes (grade IV glioma)	*MGAT1*	Mannosyl (Alpha-1,3-)-Glycoprotein Beta-1,2-N-Acetyl-glucosaminyltransferase
	RAB32	Ras-Related Protein Rab-32
	ANKRD13B	Ankyrin Repeat Domain-Containing Protein 13B
	SBK1	SH3 Domain Binding Kinase 1
	SGMS2	Sphingomyelin Synthase 2
	PLBD1	Phospholipase B Domain Containing 1
	LRRTM2	Leucine Rich Repeat Transmembrane Neuronal 2
	FSD1	Fibronectin Type III And SPRY Domain Containing 1
	WIPI1	WD Repeat Domain, Phosphoinositide Interacting 1
	BICC1	Bicaudal C Homolog 1
	SOX8	SRY (Sex Determining Region Y)-Box 8
	RRAS	Related RAS Viral (R-Ras) Oncogene Homolog
	LMF1	Lipase Maturation Factor 1
	GATAD2B	GATA Zinc Finger Domain Containing 2B
